# **Tianeptine Affects the Improvement of Behavioral Defects, such as** Schizophrenia, Caused by Maternal Immune Activation in the Mice Offspring

**DOI:** 10.2174/1871524923666230905142700

**Published:** 2023-10-23

**Authors:** Kooseung Jung, Jun-Tack Kwon

**Affiliations:** 1 Department of Clinical Pharmacology, College of Medicine, Soon Chun Hyang University, Cheonan, Republic of Korea

**Keywords:** Maternal immune activation, neurodevelopmental disorder, psychiatric disorder, tianeptine, animal model, behavioral test

## Abstract

**Background:**

Simultaneously with studies on animal models of fetal-induced maternal immune activation, related studies documented behavior, neurophysiological, and/or neurochemical disorders observed in some neuropsychiatric disorders, including autism and schizophrenia.

**Objective:**

To investigate whether treatment tianeptine might ameliorate maternal immune activation (MIA)-induced behavioral deficits in the offspring.

**Materials and Methods:**

The pregnant mice were injected through tail vein injection at a concentration of 5 mg/kg of polyriboinosinic-polyribocytidilic acid (polyI:C) and/or used saline as a vehicle. The injection was performed on the 9^th^ day of pregnancy. Each group of MIA offspring was subjected to vehicle, clozapine, or tianeptine treatment.

**Results:**

In prepulse inhibition (PPI) test, oral treatment with tianeptine ameliorated MIA-induced sensorimotor gating deficit. Most behavioral parameters of social interaction test (SIT), forced swimming test (FST), and open field test (OFT) were significantly changed in the MIA offspring. Tianeptine treatment significantly recovered behavioral changes observed in the SIT, OFT, and FST. In order to confirm expression level of neurodevelopmental proteins, immunohistochemical image analysis and Western blot were performed, and the medial prefrontal cortex (mPFC) was targeted. As a result, it was confirmed that the neurodevelopmental proteins were decreased, which was recovered after administration of tianeptine to MIA offspring.

**Conclusion:**

Tianeptine might be useful for treating psychiatric disorders with neurodevelopmental issues.

## 1 INTRODUCTION

Tianeptine is an atypical antidepressant that is mainly used for anxiety and major depressive disorder. Recently, it has also been used as a nootropic to improve cognitive performance. It is known to have a unique mechanism of action. However, its exact action mechanism has not been fully understood yet [1]. Regarding the neuroprotective action of tianeptine, it has been shown that it possesses anti-apoptotic action following 28 days of oral treatment at a dose of 50 mg/kg/day in a psychosocial stress model of tree shrews [2]. It can also prevent morphological changes in the brains of chronic stress-induced rats [3]. Other reports related to chronic stress showed that tianeptine was effective in hippocampal plasticity. It prevents the stress-induced reconstruction of glutamic synaptic vesicles in fibers adjacent to CA3 neurons, in the hippocampus area, and reverses spatial memory dysfunction caused by stress [4].

The beneficial effect of tianeptine in the above-mentioned models has been explained by its anti-inflammatory properties [4]. It has also been confirmed that tianeptine has a normalizing effect on the production of anti- and pro-inflammatory cytokines in rat hypothalamus after lipopolysaccharide treatment [5]. Recently, it has been shown that tianeptine can protect neurons and glial cells. Studies performed on astroglial cell lines have demonstrated that tianeptine can attenuate apoptosis induced by glycoprotein 120 [6]. Maternal immune activation (MIA) due to infection during the pregnancy period has been repeatedly reported to the one of the factors contributing to the etiology of neuropsychiatric developmental disorders such as bipolar disorder, autism, and schizophrenia [7-9]. To induce infection, the fetus may be exposed to MIA due to polyI:C (viral mimetic double-stranded RNA polyriboinosinic-polyribocytidilic acid) injected into the tail vein of the pregnant mice. A pro-inflammatory cytokine inducer, once or more on a prenatal day [10]. Interestingly, a wide range of behavioral disorders observed in adult mouse offspring affected by immune activation during pregnancy are also generally observed in patients with schizophrenia. These impairments include deficits in prepulse inhibition (PPI) of acoustic startle social interaction and brain abnormalities [11]. Among behavioral outcomes analyzed after prenatally MIA injection, PPI can measure sensorimotor gating function. Subsequently, functional deficiency was observed to be the same in acute/chronic patients with schizophrenia [12]. Through experimental investigation of the MIA-induced model, that is *in-vivo* rodent models, it has provided considerable evidence of psychosis-related dysfunctions in schizophrenia. Picosis-related behavioral abnormalities have shown pathological significance in terms of neurodevelopmental aspects of schizophrenia in adult life [10]. Potential effects of maternal treatments with some products, including clozapine, haloperidol, risperidone, paliperidone, and fluoxetine, on grown-up MIA offspring mice have been determined. These products have shown protective effects against the emergence of MIA offspring's behavior and structural abnormalities [13-15]. Chronic administration of clozapine has been reported to improve cognitive function in mice exposed to poly I:C for induction of MIA [14]. In addition, peri-adolescent risperidone can prevent most neuropathological changes in the MIA model [13]. Our previous work also demonstrated that MIA can induce abnormal behavior associated with neurodevelopmental protein changes in offspring. [15]. Our study has also suggested that clozapine and *Valeriana fauriei* root extract treatment could recover MIA-induced behavioral impairment in a schizophrenia animal model [15]. Our recent study showed that oral administration of red panax ginseng extract may improve behavioral impairment and increase the expression of neurodevelopmental proteins in adulthood in MIA offspring [16]. The objective of the present study was to determine whether repeated tianeptine administration could reverse behavioral deficits induced by maternal poly I:C treatment. First, we investigated behavioral symptoms by performing more than three behavioral tests with an MIA model compared with a normal control model. Second, we investigated expression levels of neurodevelopmental proteins in the medial prefrontal cortex (mPFC) of a MIA model. Furthermore, we also investigated the potential beneficial effects of tianeptine on MIA-induced offspring based on schizophrenia-like behavior and protein expression levels in mPFC. To the best of our knowledge, this is the first study to examine the effect of repeated tianeptine treatment on behavioral impairments induced by prenatal exposure to an infectious agent.

## 2 MATERIALS AND METHODS

### 2.1 Animals

C57BL6/J mice at the age of 8 weeks were purchased from Daehan BioLink Co. Ltd. (Eumseong, Korea). Mice were mated with a male-to-female ratio of 1:2 (Females, n = 20; Males, n = 10). Animal care and mate were performed as previously described [17, 18]. Procedures of animal experiments were approved by the Institutional Animal Care and Use Committee of Soonchunhyang University (Approval No. SCH16-0064).

### 2.2 Manufacture of MIA Agents and the use of Drugs

Pregnant mice were subjected to either a single injection of poly I:C (n = 12) or control (CON) (saline, n = 6) solution *via* an intravenous route on the tail under a mild physical constraint on gestational day (GD) 9. Poly I:C (potassium salt) was purchased from Sigma-Aldrich (CAS. P1530, Buchs, St Gallen, Switzerland) and dissolved in 0.9% NaCl solution. All animals were returned to new clean cages immediately after the injection and left undisturbed until the weaning of the offspring. Clozapine (CAS. C6305, Sigma-Aldrich, Seoul, Korea) was dissolved in 0.1 N HCl. Tianeptine (Sigma-Aldrich, Seoul, Korea) was dissolved in 0.9% NaCl as a high-dose stock. Clozapine and tianeptine were orally administered (drug dose: 5 mg/kg/daily and 10 mg/kg/daily once, respectively) on postnatal day 35 until postnatal day 65 (for 4 weeks) [18]. All drugs were prepared in concentrated doses and diluted to the required capacity at least one day before being used. All groups contained litters of 2 to 15 pups. The offspring were weaned after postnatal 21 days and moved group-housed. We selected male offspring for use in further experiments. Four experimental groups were tested as adults: 1) ‘control’ (n = 12 male offspring) group, animals were offspring of unstressed mothers; 2) poly I:C injected group ‘MIA’ (n = 10 male offspring), animals were offspring of mothers injected with polyI:C on pregnant day 9; 3) CLZ group (n = 10), animals were injected with poly I:C followed by CLZ administration during adolescent period; and 4) TIA group (n = 10 male offspring), animals were subjected to TIA administration during adolescent periods after injection of poly I:C.

### 2.3 Behavioral Tests

Behavioral tests were performed using animals at 10 weeks of age. All following tests used male offspring. The number of animals in each experimental group for each test was 10.

#### 2.3.1 Prepulse Inhibition (PPI)

A startle reflex system (SR Lab, San Diego Instruments, San Diego, CA, USA) was used to measure prepulse inhibition about the startle response as described previously (n = 10 animals/group). For testing, mice were gently placed into the startle response apparatus and allowed for a 5-minute acclimation period to background noise of 65 dB [18]. Trials were presented in pseudorandom order, including 14 trials with 120-dB noise for 40 ms, 30 trials in which the onset of a 20 ms broadband noise prepulse preceded the onset of a 120 dB pulse for 100 ms, and 8 trials of non-stimulus consisting of only background noise (68 dB) as previously described [14, 15].

#### 2.3.2 Forced-swim Test (FST)

Mice (n = 10 animals/group) were placed in a large transparent cylinder filled with fresh tap water (25 ± 2°C) for 5 min. Their behaviors of swimming, climbing, and immobility were recorded as described previously [16, 18]. Test scores for swimming behaviors (horizontal movement throughout the chamber and crossing quadrants), climbing behavior (upward-directed movements up the side of the chamber and jump-ups from the bottom of the chamber), and immobility (no additional activity other than keeping the head above water or tiny whip kicks) were recorded.

#### 2.3.3 Open-field Test (OFT)

Mice (n = 10 animals/group) were removed from their home cages and placed individually. The open field test was performed in the center zone (17 × 17 cm) of an open field arena (50 × 50 cm) for 20 min as described previously [16, 18].

#### 2.3.4 Social Interaction Test (SIT)

SIT was performed as described previously. Social interaction partners of mice (n = 10 animals/group) had the same gender with similar body weights. The social test was performed in a darkened environment, which was maintained until the test ended. For this test, a single bulb of 25 W red light was used to illuminate the test chamber. The bulb was placed not over 100 cm above the chamber bottom. The test was performed for 20-min for each pair [16, 18].

### 2.4 Western Blot

Medial prefrontal cortex (mPFC) tissues (n = 5~6 animals/group) were lysed with radioimmunoprecipitation assay (RIPA) buffer of ELPIS-Biotech containing protease inhibitors including 1 mM PMSF and centrifuged at 18,341 x g for 10 min at 4°C. Lysed proteins (80 µg for each sample) were subjected to 10 and 12% sodium dodecyl sulfate-polyacrylamide gel electrophoresis (SDS-PAGE) and transferred to polyvinylidene difluoride (PVDF) membranes (Millipore company). These membranes were probed with the following primary antibodies: anti-Dpysl2 (dihydropyrimidinase-like 2, #9393, 1:1,000; Cell Signaling Technology, Danvers, MA, USA), anti-Lasp1 (LIM and SH protein 1, MAB8991, 1:2,000; Millipore, Billerica, MA, USA), anti-Nefm (neurofilament M, #2838, 1:1,000; Cell Signaling Technology, Danvers, MA, USA), and anti-Tubb (β-tubulin; 1:3,000; Thermo Fisher Scientific, Inc., Rockford, IL, USA). Subsequently, these membranes were probed with either an anti-mouse IgG (A9044, 1:10,000; Sigma. St. Louis, MO, USA) or anti-rabbit IgG (LF-SA8002, 1:5000; Abfrontier, Seoul, Korea) secondary antibody as described previously [18].

### 2.5 Immunohistochemistry

Mice (n = 4~5 animals/group) were anesthetized with ethyl ether and perfused with 1x PFA buffer containing 4% paraformaldehyde as described previously. Frozen sections were blocked with normal horse serum of Vector Laboratories, Inc (S-2000, Burlingame, CA, USA) and incubated with anti-dpysl2 (HPA002381, 1:700; Atlas Antibodies AB, Stockholm, Sweden), Nefm (#2838, 1:100; Cell Signaling Technology, Danvers, MA, USA), and anti-NeuN (MAB377, 1:100; Millipore, Billerica, MA, USA) antibodies at 4°C overnight. They were then incubated with Cy3-conjugated anti-rabbit and anti-mouse IgG secondary antibodies (Jackson company, #111-165-003, 1:500 and #715-545-151, 1:800), Thermo Fisher Scientific, Inc., Rockford, IL, USA). Subsequently, these membranes were probed with an anti-mouse IgG (A9044, 1:10,000; Sigma. St. Louis, MO, USA) or anti-rabbit IgG (LF-SA8002, 1:5000; Abfrontier, Seoul, Korea) secondary antibody as described previously [18].

### 2.6 Statistical Analysis

Data are presented as mean ± standard error of the mean (SEM). All data were analyzed using one-way analysis of variance (ANOVA) followed by the Tukey post hoc test for verification. All statistical analyses were performed using SPSS 23.0 (SPSS Inc., Chicago, IL, USA). A statistically significant difference was considered at *p* < 0.05 unless otherwise indicated.

## 3 RESULTS

### 3.1 Prepulse Inhibition

**Fig. (1):**
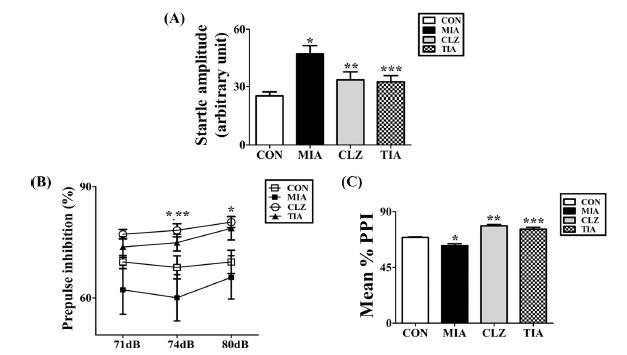
Modulation of PPI of ASR by preadolescent tianeptine treatment in offspring born to poly I:C-treated and control mothers. (**A**) This figure depicts startle reactivity to prepulse-alone trials as a function of prepulse intensity. (**B**) Inhibition of startle reflex to a 120-dB. A stimulus was achieved through prepulse of distinct intensities into 3, 6, and 12 dB above a background of 68 dB. (**C**) PPI levels were calculated using the following formula: mean PPI = 100 - {[(startle response for prepulse + pulse trial) / (startle response for pulse alone trial)] × 100}. The mean %PPI is depicted for all experimental groups. Data are presented as mean ± SEM (*p < 0.05 compared to CON, **p < 0.05 compared to MIA, ***p < 0.05 compared to MIA). **Abbreviations:** PPI: prepulse inhibition; ASR: acoustic startle response; CON, offspring of non-MIA mice; MIA, offspring of MIA mice; CLZ, intraperitoneal injection with clozapine of Poly I:C-injection group; TIA, intraperitoneal injection with tianeptine of Poly I:C-injection group.

The prepulse inhibition test was performed to measure sensorimotor gating function, a filtering mechanism to protect the brain from overstimulation. This function has consistently been found to be reduced in individuals with schizophrenia investigated by prepulse inhibition test [12]. In this study, the MIA model shows reduced PPI while tianeptine could reverse this deficit. To determine the treatment effect of tianeptine on the function of sensorimotor gating, we performed acoustic startle response (ASR) and PPI analysis for mice in each of the following groups: 1) control mice, 2) MIA-induced mice with PPI disruption, and 3) tianeptine treated MIA mice. One-way ANOVA test showed that tianeptine significantly decreased ASR levels and reversed PPI disruption caused by MIA induction as shown in Fig. (**1**). PPI values were reduced at prepulse levels of 71, 74, and 80 dB in the MIA-induced group compared with those of the control group (Fig. **1B**). MIA-induced PPI disruption was consistently observed at all prepulse levels (71, 74, and 80 dB) in 120-dB pulse stimulus trials. Mean % PPI levels in MIA-induced offspring were significantly (*p* < 0.05) decreased compared to those in normal control offspring (Fig. **1C**). This indicated that sensorimotor gating functional deficits were induced by MIA. Moreover, disturbance of PPI caused by MIA induction was significantly (*p* < 0.05) improved after treatment with an atypical antipsychotic (clozapine) and an antidepressant (tianeptine).

### 3.2 Forced Swim Test

**Fig. 2:**
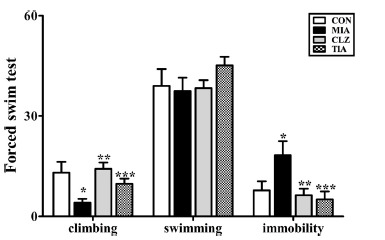
Data are presented as mean ± SEM (*p < 0.05 compared to CON, **p < 0.05 compared to MIA, ***p < 0.05 compared to MIA). **Abbreviations:** CON, offspring of non-MIA mice; MIA, offspring of MIA mice; CLZ, intraperitoneal injection with clozapine of Poly I:C-injection group; TIA, intraperitoneal injection with tianeptine of Poly I:C-injection group.

There were significant differences in the results of FST among control, MIA, clozapine-treated MIA (CLZ), and tianeptine-treated MIA (TIA) groups (Fig. **2**). MIA offspring exhibited significantly (*p* < 0.05) decreased climbing behaviors compared to those of the control group. The climbing function decreased in the MIA group was significantly (*p* < 0.05) increased after clozapine or tianeptine treatment (Fig. **2**). The number of occurrences of immobility was significantly (*p* < 0.05) increased in MIA offspring compared with that in control offspring. MIA offspring showed a higher number of occurrences than control mice. Changed immobile behaviors were recovered significantly (*p* < 0.05) in the TIA group and the CLZ group.

### 3.3 Open-field Test

Numbers of rearing, cage sniff, immobility, and duration of rearing and immobile behaviors were significantly (*p* < 0.05) decreased in the MIA group than in the control group. Most scores were improved to normal levels after clozapine or tianeptine treatment (Table **1**). The number of central entries and line crossing was significantly (*p* < 0.05) decreased in the MIA group compared to that in the control group. The score was recovered (*p* < 0.05) to normal control level after clozapine or tianeptine treatment (Table **1**). The number and duration of rearing behavior were decreased (*p* < 0.05) in the MIA group compared to that in the control group. The number of rearing was not significantly (*p* > 0.05) recovered by drug treatment (Table **1**). However, the duration score of rearing was reversed in the CLZ group. The scores of MIA group behavior were increased in the number and duration of immobility compared to the control group (*p*<0.05). These scores were significantly (*p* < 0.05) recovered to normal control levels after clozapine or tianeptine treatments (Table **1**).

### 3.4 Social Interaction Test

The MIA offspring showed several social deficits (Table **2**). The number and duration of non-aggressive behavior (cage sniff) were significantly (*p* < 0.05) decreased in the MIA group compared to those in the control group (Table **2**). These scores were increased to normal control levels after treatment with clozapine (number of sniffing) or tianeptine (duration of sniffing). Aggressive behaviors (aggressive grooming and biting) except fighting during SIT were significantly changed in the MIA group compared to those in the control (Table **2**, *p* < 0.05). These scores were recovered to normal levels after drug treatment. The number and duration of aggressive grooming were recovered (*p* < 0.05) after treatment with clozapine, but not after treatment with tianeptine (Table **2**). The number and duration of biting behavior were significantly (*p* < 0.05) increased in the MIA group compared to the control group. These scores were decreased (*p* < 0.05) to normal control levels in all drug treatment groups (CLZ and TIA) (Table **2**). These results indicated that MIA-induced social interaction impairment could be reversed (*p* < 0.05) by treatment with tianeptine as an antipsychotic (Table **2**).

### 3.5 Western Blot & Immunohistochemistry

**Fig. (3):**
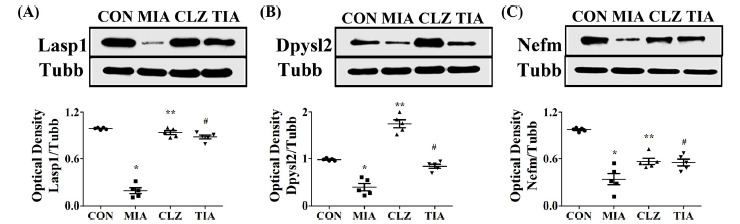
Expression levels of LIM and SH3 protein 1 (Lasp1), dihydropyrimidinase-like 2 (Dpysl2), and neurofilament M (Nefm) in brains of MIA-induced mice by Western blot analysis. Nefm, Lasp1, Dpysl2, and Dlg4 expression levels were determined by western blot analysis. Actb was used as an internal control. Expression levels of Nefm, Dpysl2, Lasp1, and Dlg4 proteins in the medial prefrontal cortex of the brain were decreased in the MIA group compared with those in the normal control. Decreased expression levels of Nefm, Lasp1, Dpysl2, and Dlg4 were recovered after treatment with PG. Quantitative analysis of (**A**) Lasp1 expression, (**B**) Dpysl2 expression, and (**C**) Nefm expression. Data are presented as mean ± SEM (*p < 0.05 compared to CON, **p < 0.05 compared to MIA). **Abbreviations:** CON, offspring of non-MIA mice; MIA, offspring of MIA mice; CLZ, intraperitoneal injection with clozapine of Poly I:C-injection group; TIA, intraperitoneal injection with tianeptine of Poly I:C-injection group.

**Fig. (4):**
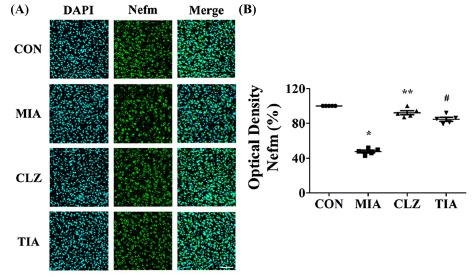
Immunohistochemical analysis of neurofilament M (Nefm) expression in MIA-induced mice brains. (**A**) An immunofluorescent staining image of the medial prefrontal cortex (mPFC) was obtained from a confocal microscope. Nefm was stained with Alexa Fluor 488 (green color) for meddle size of neurofilaments. Nuclei were stained with DAPI (blue color). Fluorescent staining revealed a decrease of Nefm in the MIA group compared to that in the control. Scale bar, mPFC, 50 µm. **(B**) Quantitative analysis of Nefm expression. Data are presented as mean ± SEM (*p < 0.05 compared to CON, **p < 0.05 compared to MIA). **Abbreviations:** CON, offspring of non-MIA mice; MIA, offspring of MIA mice; CLZ, intraperitoneal injection with clozapine of Poly I:C-injection group; TIA, intraperitoneal injection with tianeptine of Poly I:C-injection group.

**Fig. (5):**
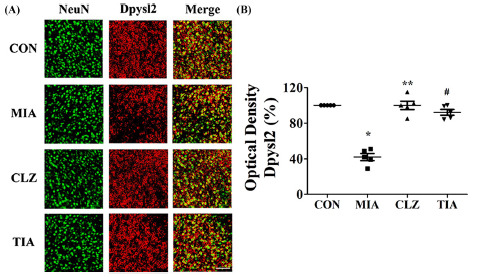
Immunohistochemical analysis of dihydropyrimidinase-like 2 (Dpysl2) expression in MIA-induced mice brains. (**A**) An immunofluorescent staining image of the medial prefrontal cortex was obtained with a confocal microscope. Dpysl2 was stained with Cy3 (red color). NeuN was stained with Alexa Fluor 488 (green color). Nuclei were stained with DAPI (blue color). Fluorescent staining revealed a decrease of Dpysl2 in the MIA group compared to that in the control. Scale bar, mPFC, 50 µm. (**B**) Quantitative analysis of Dpysl2 expression. Data are presented as mean ± SEM (*p < 0.05 compared to CON, **p < 0.05 compared to MIA). **Abbreviations:** CON, offspring of non-MIA mice; MIA, offspring of MIA mice; CLZ, intraperitoneal injection with clozapine of Poly I:C-injection group; TIA, intraperitoneal injection with tianeptine of Poly I:C-injection group.

Mice prenatally exposed to Poly I:C showed altered expression levels of neurodevelopmental proteins. Western blot analyses were performed to determine whether there were changes in expression levels of neurodevelopmental proteins such as Nefm, Lasp1, and Dpysl2 in MIA offspring (Fig. **3**). Results showed that levels of these three proteins in mPFC were significantly (*p* < 0.05) lower in MIA offspring than in control offspring (Fig. **3**), although expression levels of Tubb in mPFC were similar among all groups. These changes were restored (*p* < 0.05) by clozapine or tianeptine treatment (Figs. **3A** and **3C**). Despite this increase, it could not be concluded that the medication control was directly induced by clozapine or tianeptine because it was not included in this study. However, clozapine and tianeptine have been shown to have at least a partial effect on protein expression levels. To determine whether proteins of neurodevelopmental such as Nefm, Lasp1, and Dpysl2 were regulated in the MIA offspring, we also performed immunohistochemical staining using mPFC areas of brain tissues from control, MIA, CLZ, and TIA mice. Results are shown in Figs. (**4** and **5**). Nefm and Dpysl2 were differentially expressed in immunofluorescent-stained brain images of CON, MIA, CLZ, and TIA groups, showing significant (*p* < 0.05) differences in immunohistochemical staining intensities (Figs. **4** and **5**). Results revealed significant increases of these proteins (all *p* < 0.05). Protein levels of Nefm and Dpysl2 were also different in immunofluorescent-stained brain images among the four groups. Their intensities in immunohistochemical staining images showed significant differences (*p* < 0.05, Figs. **4B**, **5B**).

## 4 DISCUSSION

This study showed that exposure to MIA during pregnancy affects schizophrenia-like behavior in adult progeny mice, along with decreased sensory motor function and increased astounding sensitivity. These behavioral disorders caused by MIA exposure were recovered after treatment with Tianeptine of antidepressant drug or Clozapine of antipsychotic drug. Results of Western blot and immunohistochemical analysis showed that the levels of several neurodevelopmental protein expressions of mPFC tissues in MIA offspring were downregulated compared to the control group. This downregulation was recovered after treatment with tianeptine. To the best of our knowledge, this is the first report showing that treatment with the antidepressant Tianeptine can restore behavioral changes and increase neurodevelopmental proteins in MIA-induced schizophrenia animal models. Exposure to inflammation and/or infection during pregnancy can induce an immunological lesion in the developing brain. It has been reported that the fetal brain shows abnormal fetal expression of pro-inflammatory cytokines and activated microglia upon exposure to virulent pathogens. During the early fetal brain development period, exposure to infection-induced disruption can significantly affect the postnatal brain, subsequently affecting development and maturation. It can also lead to functional and structural deficits depending on postnatal maturation processes. Hence, immune activation models of prenatal exposure should include developmental aspects of abnormal brain function and structure in adulthood [19]. The feature of an immune activation model of prenatal exposure can be particularly relevant to the neurodevelopmental perspective of schizophrenia because the pathophysiological and neuropathological mechanisms of schizophrenia are assumed to be progressive in nature [10]. Tianeptine is an atypical antidepressant agent with a novel neurochemical and comparatively favourable pharmacokinetic profile. It can increase serotonin (5-HT) uptake and reduce stress-induced atrophy of neuronal dendrites in the brain. Tianeptine has shown a low propensity for abuse. It does not appear to be associated with adverse psychomotor, cognitive, cardiovascular disorders, sleep deficit, or body-weight effects. In contrast with selective serotonin reuptake inhibitors (SSRIs) and most tricyclic antidepressant agents, tianeptine might have additional properties to show serotoninergic effects in synapses and various neuronal systems. Tianeptine has a wide profile of action. It has effects on neuroplasticity and neuroprotection in the hippocampal region. It also shows anticonvulsant and antinociceptive efficacy [20-23]. Regarding its neurobiological properties, tianeptine shows modulatory effects on the activity of the hypothalamus-pituitary-adrenal axis and symptoms of sickness behavior induced by LPS or interleukin-1 (IL-1) injection [24]. Based on these studies, tianeptine as an atypical antidepressant drug might modulate processes of inflammation in the brain, further contributing to its therapeutic efficiency for depressed patients. Our previous study has shown that Panax ginseng (PG) could be used as a candidate to treat several symptoms of schizophrenia in adult offspring with prenatal exposure to poly I:C. In the present study, MIA induction increased aggressive behaviors including fighting and biting. However, such behaviors were restored by oral treatment with tianeptine. FST and OFT results showed that some behavioral patterns indicating depressive behaviors were decreased by tianeptine treatment. Moreover, it has been shown that expression levels of neurodevelopmental proteins are increased in the MIA model after PG application [16]. It has also been demonstrated that PG could be potentially used to treat various psychological disorders as a natural alternative remedy. Several studies have suggested that PPI impairment of astounding responses in both animal models and schizophrenic patients may reflect impairment of sensory movement [16, 25, 26]. This study showed that MIA-induced PPI deficiency can be significantly reversed by treatment with tianeptine, similar to the effectiveness of atypical antipsychotics. Impaired social interaction behavior was observed in mice exposed to MIA, which can lead to negative/cognitive symptoms of schizophrenia, especially including social withdrawal, cognitive dysfunction, and apnea. It can cause behavioural disorders in adulthood [10, 11, 16]. These results have expanded our previous observations showing that prenatal exposure to poly-I:C mice can induce aggressive behaviors in SIT [16]. In the present study, we determined expression levels of Nefm and Dpysl2 proteins. Our results suggested that the application of a repeated variable MIA during critical periods of fetal brain development could result in changes in expression levels of neurodevelopmental proteins such as Nefm and Dpysl2. As a microtubule-binding protein, Dpysl2 plays a regulatory and structural role in cytoskeletal dynamics, synaptic transmission, and vesicle tracking in the developing brain [27, 28]. Furthermore, altered Dpysl2 activity is related to schizophrenia in an animal model. Polymorphisms of its genes are associated with susceptibility to schizophrenia [27]. We presented the therapeutic effects of tianeptine as an antidepressant and clozapine as an antipsychotic in an MIA model. Tianeptine works by decreasing extracellular levels of serotonin without decreasing the release of serotonin in rat brains [29]. Clozapine is a synthetic dibenzodiazepine derivative and an atypical antipsychotic agent. It inhibits several neurotransmitter receptors in the brain [14]. Moreover, clozapine is related to functions of sensorimotor gating that can be restored in mean% PPI and/or PPI scores following clozapine treatment in an MIA-induced schizophrenia animal model [14]. Although clozapine and tianeptine have different mechanisms, our results of western blot and immunohistochemical analyses showed that down-regulation of several neurodevelopmental proteins could be recovered in the prefrontal cortex of the MIA model following treatment with these two drugs. This might explain why behavioral changes in the MIA model were restored after treatment with tianeptine or clozapine. In our study, we focused on the effects of clozapine and tianepine in restoring neurodevelopmental deficits induced by MIA without focusing on the exact mechanisms involved. Clozapine, an antipsychotic drug, was used as a positive control for MIA-induced schizophrenia animal model based on previous studies [14, 15, 18]. Therefore, the exact reason why the effects of tianeptine and clozapine on MIA offspring are similar is currently unclear. In schizophrenic subjects, changes in volumes and neuronal atrophy in different brain regions are important hallmarks. For example, schizophrenia subjects commonly show ventricular enlargement [30] with decreased spine density and soma size of prefrontal cortex neurons [31]. Additionally, heterozygous reeler and ErbB2/B4-CNS knock-out mice models show impaired dendrite spines and behavioral abnormalities that might be affected by perturbation of glutamatergic synapses associated with schizophrenia-like symptoms in various brain regions [32-34]. Dendritic spine pathology is thought to be related to the pathology of schizophrenia [35]. Many studies have reported that dendritic spine density is decreased in multiple brain regions such as the neocortex [36], the striatum [37], and the hippocampal formation [38] of patients with schizophrenia. Clozapine treatment might decrease hyperresponsiveness in a novel environment by decreasing spinogenesis and neuronal rearrangement in neurons of the nucleus accumbens. Critchlow *et al*. (2006) have reported that clozapine can increase synaptogenesis and synaptic maturation *in vitro* in rats [39]. Neurofilaments can functionally maintain neuronal caliber to form part of the axon skeleton. They might also play a role in intracellular transport to dendrites and axons. One study on pyramidal neurons based on distribution and expression in the prefrontal cortex has found that subunits are altered in mood disorders and schizophrenia [40]. The present study also investigated expression levels of proteins for neurodevelopment based on two previous reports [16, 18]. Our results revealed that the application of MIA during critical development periods of the fetal brain could result in changes in expression levels of neurodevelopmental proteins such as Dpysl2 and neurofilament proteins. These proteins might have enduring effects on axonal outgrowth and synaptic function in the offspring later during adolescence to adulthood [27]. Decreases in protein expression of Nefm and Dpysl2 due to MIA were affected by clozapine or tianeptine oral treatment. Therefore, these proteins might be involved in abnormal symptoms and behaviors of MIA mice indirectly and/or directly. Results of the present study showed an additional role of tianeptine as an antidepressant in the pathogenesis of psychiatric disorders such as maternal inflammation-induced schizophrenia. Many studies of mental disorders and schizophrenia-related disorders have focused on the effects of antipsychotics such as clozapine in the fetal period and its side effects in adolescents and/or early adults. In contrast, we investigated the effect of tianeptine as one antidepressant in a model geared toward adolescents and early adults by analyzing expression levels of neurodevelopment proteins and changing behavioral patterns.

## CONCLUSION

Our results suggest that tianeptine may be useful as a treatment option for schizophrenia. The inhibition of psychosis might be achieved through the pharmacological effect of tianeptine. However, this model, shown in our study, mainly causes some cognitive defects, especially improvement behavior defects associated with some of the schizophrenia symptoms associated with attention. Thus, this model does not fully reflect the complexity of schizophrenia, and this model provides limited value for understanding the pathology of the disease.

## Figures and Tables

**Table 1:** Behavior of adolescent mice in CON, MIA, MIA + CLZ, and MIA + TIA groups during an open field test.

**Behaviors**	**CON**	**MIA**	**MIA+CLZ**	**MIA+TIA**
**Number of Behaviors (N)**				
Central entered	30.9 ± 3.0	17.1 ± 2.1	22.4 ± 2.6	24.6 ± 3.8
Line crossing	9.7 ± 0.8	4.8 ± 0.9	5.9 ± 0.8	6.1 ± 1.2
Run	0.1 ± 0.1	0.9 ± 0.4	0.1 ± 0.1	0.1 ± 0.1
Rear	116.0 ± 4.0	79.3 ± 6.9a	91.6 ± 3.7	83.4 ± 4.1
Grooming	7.0 ± 0.8	5.6 ± 1.1	7.2 ± 0.9	7.2 ± 0.9
Cage sniff	174.3 ± 8.1	141.7 ± 11.3a	161.3 ± 4.7b	161.3 ± 4.7c
Immobility	3.2 ± 1.0	53.1 ± 13.6a	4.6 ± 0.7b	3.2 ± 0.8c
**Duration of Behavior (Sec)**				
Run	0.2 ± 0.2	1.4 ± 0.6	0.2 ± 0.2	0.2 ± 0.2
Rear	239.2 ± 10.6	153.2 ± 15.0a	198.9 ± 7.9	201.3 ± 11.1
Grooming	43.4 ± 3.1	28.1 ± 6.0	141.3 ± 4.7b	42.4 ± 7.7
Cage sniff	503.9 ± 30.8	484.4 ± 31.8	556.6 ± 36.7b	547.2 ± 36.8
Immobility	7.7 ± 2.5	135.7 ± 46.3a	8.7 ± 1.6b	5.8 ± 1.3c

**Note: **^a^*p* < 0.05 *vs*. CON; ^b^ *p* < 0.05 *vs*. MIA; Data are presented as mean ± standard error of the mean. **Abbreviations:** CON, control group; MIA, maternal induced activation group; n, number of behaviors observed; MIA+CLZ, clozapine-treated MIA group; MIA+TIA, tianeptine-treated MIA group.

**Table 2:** Behavior of adolescent mice in CON, MIA, MIA + CLZ, and MIA + TIA groups during a social interaction test.

**Behaviors**	**CON**	**MIA**	**MIA+CLZ**	**MIA+TIA**
**Number of Behaviors (N)**				
Sniff	117.7 ± 3.17	37.5 ± 3.51a	84.44 ± 5.83b	84.78 ± 6.02c
Follow	3.7 ± 0.98	20.3 ± 6.78a	5.89 ± 1.18b	6.33 ± 1.36c
Grooming to the partner	1.2 ± 0.44	0.3 ± 0.21	0.11 ± 0.11	2.33 ± 1.62
Fight	0.20 ± 0.13	0.60 ± 0.34	0.22 ± 0.22	0.00 ± 0.00
Aggressive grooming	0.1 ± 0.1	1.2 ± 0.47	0.22 ± 0.22	0.33 ± 0.24
biting	2.8 ± 0.73	10.5 ± 1.17	0.11 ± 0.11b	0.00 ± 0.00c
**Duration of Behavior (Sec)**				
Sniff	158.7 ± 5.02	94.2 ± 5.66a	172.11 ± 19.24b	94.56 ± 7.89
Follow	9.9 ± 2.68	7.2 ± 1.39	5.89 ± 1.18	13.22 ± 3.79
Grooming to the partner	7.1 ± 3.33	1.8 ± 1.28	0.22 ± 0.22	7.67 ± 4.35
Fight	0.20 ± 0.13	1.4 ± 0.79	0.22 ± 0.22	0.00 ± 0.00
Aggressive grooming	0.00 ± 0.00	3.4 ± 1.41	0.33 ± 0.33	1.78 ± 1.19
biting	11.3 ± 3.91	23.7 ± 5.03	4.00 ± 3.18b	0.00 ± 0.00c

**Note: **^a^*p* < 0.05 *vs*. CON; ^b^ *p* < 0.05 *vs*. MIA; Data are presented as mean ± standard error of the mean. **Abbreviations:** CON, control group; MIA, maternal induced activation group; n, number of behaviors observed; MIA+CLZ, clozapine-treated MIA group; MIA+TIA, tianeptine-treated MIA group.

## Data Availability

The data that support the findings of this study are available within the article.

## LIST OF ABBREVIATIONS

SEM: Standard Error of the Mean
ANOVA: One-way Analysis of Variance
SDS-PAGE: Sodium Dodecyl Sulfate-Polyacry-lamide Gel Electrophoresis
mPFC: Medial Prefrontal Cortex
PVDF: Polyvinylidene Difluoride
GD: Gestational Day
PG: Panax Ginseng
